# Supplementary material for: Altered Distribution and Expression of Syndecan-1 and -4 as an Additional Hallmark in Psoriasis
Source: Int J Mol Sci. 2022 Jun 10;23(12):6511. doi: 10.3390/ijms23126511 (PMC9224530; doi:10.3390/ijms23126511)
Supplement: Supplementary file 1 [file ijms-23-06511-s001.zip › ijms-1734653-supplementary.pdf]

Supplementary Figure

(a)

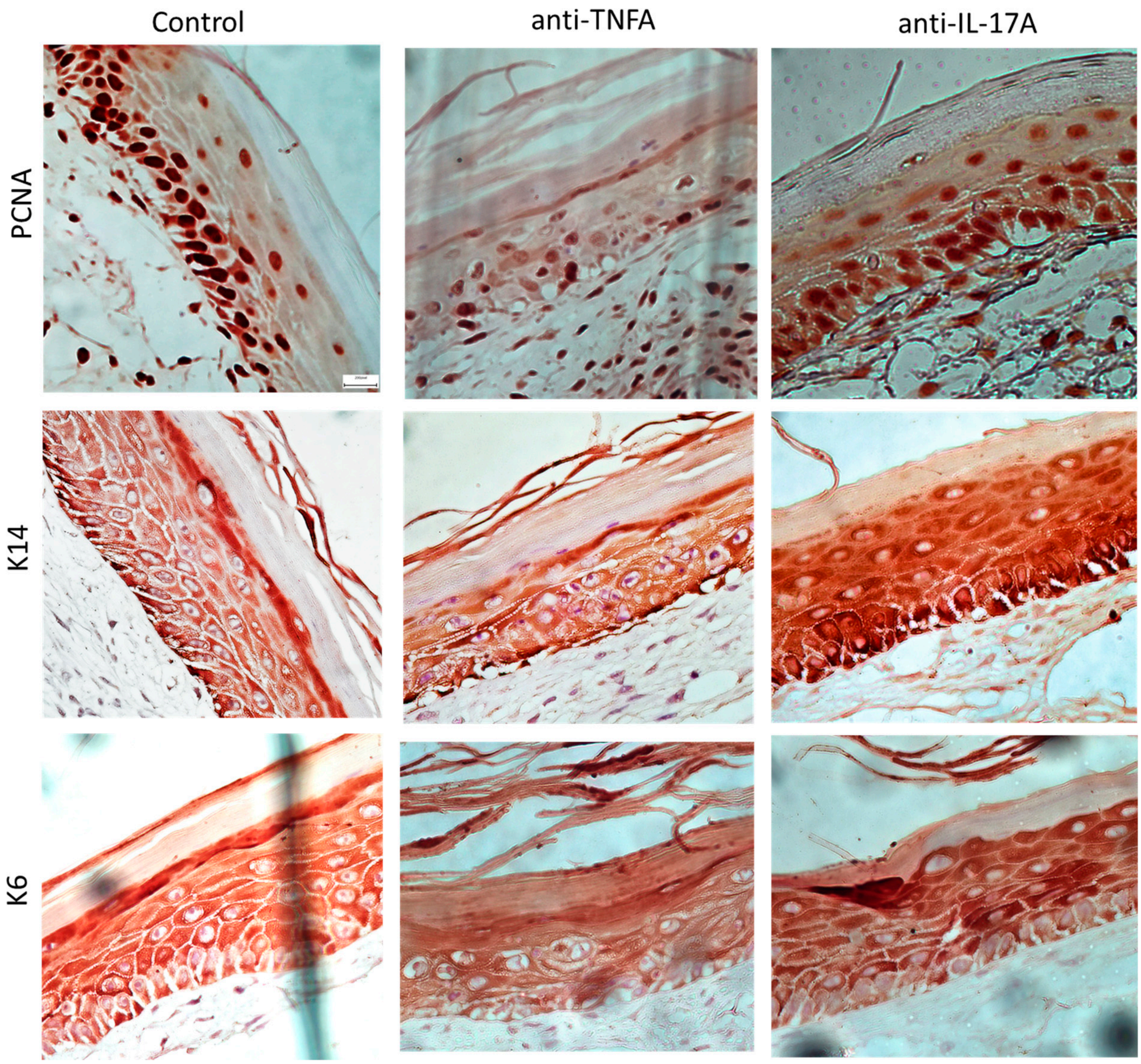

(b)

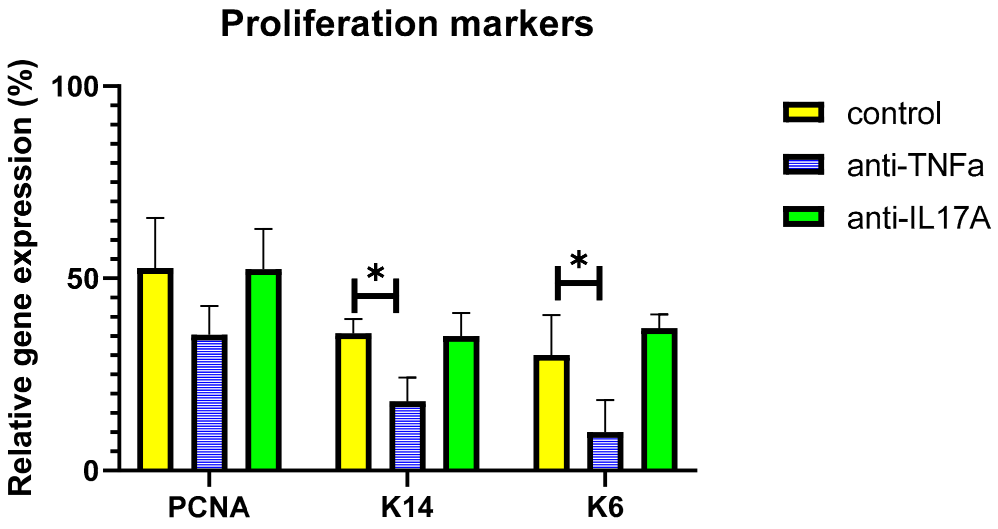

Figure S1. Proliferation activity of epidermal cells after treatment with TNFa or IL17-A blockers in a 3D full-thickness skin model. (a) Immunohistochemical staining against PCNA proliferation marker, keratin-14 as a marker of undifferentiated dividing keratinocytes in the basal epidermis and keratin-6 as a marker of "activated" keratinocytes of the upper layers. (b) Statistical analysis of staining quantification expressed as the % score of positive cells/total number of counted cells. Three images corresponding to three independent experiments were analyzed per condition. mean± SD, t-student test, *p<0,05.
